# Incorporating a non-cognitive selection method in the residency program: the complexities of effective situational judgment test item writing

**DOI:** 10.3389/fmed.2026.1747830

**Published:** 2026-03-19

**Authors:** Diantha Soemantri, Syntia Nusanti, Natalia Widiasih Raharjanti, Fitri Octaviana, Aulia Rizka, Prasetyanugraheni Kreshanti, Rita Mustika

**Affiliations:** 1 Department of Medical Education, Faculty of Medicine Universitas Indonesia, Jakarta, Indonesia; 2 Department of Ophthalmology, Faculty of Medicine Universitas Indonesia, Jakarta, Indonesia; 3 Department of Psychiatry, Faculty of Medicine, Universitas Indonesia, Jakarta, Indonesia; 4 Department of Neurology, Faculty of Medicine, Universitas Indonesia, Jakarta, Indonesia; 5 Department of Internal Medicine, Faculty of Medicine, Universitas Indonesia, Jakarta, Indonesia; 6 Cleft and Craniofacial Center Dr. Cipto Mangunkusumo Hospital, Division of Plastic Reconstructive and Aesthetic Surgery, Department of Surgery, Faculty of Medicine, Universitas Indonesia, Jakarta, Indonesia

**Keywords:** medical residency program, non-cognitive attributes, professionalism, selection, situational judgment test

## Abstract

**Introduction:**

Medical residency programs have been pivoting toward incorporating non-cognitive selection methods. Since Indonesia is accelerating its residency training program, more inclusive methods such as Situational Judgment Tests (SJTs) are preferable. As relatively early adopters of SJT, we argue for the importance on reflecting on the item writing process. This study aimed to examine the SJT item writing process for residency selection in Indonesia, specifically by mapping its content and associated features or characteristics.

**Methods:**

Twenty one subject matter experts from 11 residency programs served as item writers. After developing test specifications, the item writers used a provided template to create scenarios and corresponding response options. The analysis involved identifying the domain and content of the SJT scenarios and assessing potential biases related to gender, language and sensitivity.

**Results:**

A total of 106 SJT scenarios were developed, with commitment to professionalism as the dominant domain. The scenarios varied in length, from 21 to 145 words, and were designed to be neutral in terms of language, gender and sensitivity. Several drawbacks were identified, including excessive focus on clinical decision making and specific medical specialty contexts and limited information provided within some scenarios.

**Discussion:**

The findings revealed several pitfalls which include construct irrelevance and lacking of contextual details. A structured faculty development on SJT writing should take into account those nuances that can threaten the test’s validity. To sustain the development and further adoption of SJT as a selection method, continuous support and feedback for the item writers are crucial.

## 1 Introduction

Current evidence emphasize the need for a comprehensive selection process, one that assesses candidates not only on cognitive aspects but also on non-cognitive attributes. Papadakis et al. (1) seminal article on the correlation between professionalism lapses during medical school and subsequent malpractice events in clinical practice highlighted the importance of selecting candidates based on their future ‘potential’ for non-cognitive attributes, including professionalism. Non-cognitive attributes have been recognized as one of the elements that determine the well-functioning of practicing physicians (2). Situational Judgment Tests (SJTs) have emerged as one of the methods for selecting students based on their non-cognitive attributes.

A number of methods are commonly used to select candidates based on their non-cognitive aspects, such as traditional interviews, multiple mini interview (MMI), Minnesota Multiphasic Personality Inventory and other standardized psychological tests. However, the evidence regarding the validity of these methods is mixed. The Minnesota Multiphasic Personality Inventory focused only on future psychopathology issues such as depression and schizophrenia, not on contextual non-cognitive attributes required from physicians in their daily medical practice (3). While MMI, despite its superiority compared to traditional interviews due to its increased objectivity and standardized structure across all candidates, requires substantial amount of resources to run (4). Compared with at least the previous two methods, SJTs demonstrate several merits in terms of their cost-effectiveness and predictive validity (5).

Many countries, including those in the Global South, are grappling with a significant shortage of doctors, including specialists, both in numbers and distribution (6–8). Indonesia is not immune to this issue; its Ministry of Health recently revealed a need for an additional 70,000 specialist doctors nationwide, which might be fulfilled only after 20 years given the current annual production rate of 2,700 specialist doctors (9). In response, the country is accelerating the training of specialist doctors by empowering its medical faculties and teaching hospitals across the country. The ultimate aim of Indonesia’s acceleration program is to produce and deploy specialist doctors across all regions of the country, particularly rural and developing areas. One way to achieve this goal is to recruit more students or trainees from rural areas, so they can return to and serve their home communities upon completion. In fact, a compelling body of evidence suggests that doctors are more likely to return to rural areas if they are from those areas (10).

Therefore, any chosen selection methods should not focus only on identifying and selecting the candidates who are more likely to complete their studies and become competent general or specialist doctors (11), but must also prioritize diversity and inclusiveness (12). Gardner et al. (13) demonstrated that candidates from underrepresented minorities or underprivileged background will have higher chances to be admitted into the specialist program when SJT was used as one of the selection methods. While MMI has shown adverse impacts on student selection; students from more privileged backgrounds often have access to MMI coaching or practice, increasing their likelihood of passing the MMI compared to their underprivileged counterparts (14).

For the reasons mentioned above, SJTs are being explored as a viable alternative for the Indonesian residency program’s selection system. The SJT can be administered efficiently to a large number of candidates simultaneously and has been shown to have less adverse impacts on diversity (15), including within the Indonesian context (16). Given the national push to accelerate specialist training, establishing valid and fair selection tools such as SJT becomes even more critical, especially in developing countries such as Indonesia where resources are scarce.

To the best of our knowledge, there are no studies that examined the development and testing of SJT for residency selection in Indonesia. There have been many studies on the validity of SJTs, but less so on the process of developing one, specifically on the item writing phase. Despite emerging evidence on the positive correlation between SJT scores and other performance metrics, several studies have argued for the need for a better clarity of the actual construct SJT is assessing (2, 5, 17, 18). According to Lievens and Motowidlo (17), there are two aspects on which SJT operates. First is the specific procedural knowledge on how to work effectively in job-related situation and this requires knowledge about that particular job. The second is the more general knowledge about the costs and benefits of expressing certain traits in certain job-related situation and this knowledge may be acquired before commencing the job. However, McDaniel et al. (18) argue that these two aspects are often overlap and difficult to be distinguished when assessing responses toward SJT.

In light of the above we argue that increasing stakeholder awareness and engagement is important to initiate the SJT development process, hence establishing a pool of educators equipped with the skills to write effective SJT items is essential (19). For a resource-constrained setting, running and sustaining a structured faculty development program specifically on SJT can be a significant challenge, also given the fact that subject matter experts (SMEs) are not all inherently good item writers. In addition, we argue for the existence of cultural nuances related to non-cognitive attributes that we have to take into account when developing SJTs. The key to a robust SJT is a valid pool of items that represents the wide spectrum of non-cognitive attributes required of residents within a particular context and embodies the relevant cultural nuances (16).

The present study is aimed at analyzing the SJT content and associated features or characteristics that have been developed for the Indonesian residency selection context. Our study would like to unpack the process of developing SJTs starting from the item writing process. Understanding the nature of the SJT items developed within the Indonesian residency context can help us identify key lessons learned for the future and continuous development and use of SJTs in various other settings as well. We believe our findings would be useful for other countries or settings sharing similar struggles or challenges and at different levels of SJT adoption.

## 2 Materials and methods

The study obtained ethical clearance from the Research Ethics Committee of the Faculty of Medicine, Universitas Indonesia (No. 1364/UN2.F1/ETIK/PPM.00.02/2024).

This study used a descriptive qualitative design to map the content and analyze the associated features of SJT items for residency selection. The process began with an item writing workshop for 21 clinical teachers (15 females and 6 males) from 11 residency programs with the following specialty distribution: ophthalmology (3), forensic medicine (1), radiology (1), ob-gyn (1), internal medicine (2), anesthesiology (2), psychiatry (3), pathology anatomy (1), surgery (4), neurology (2) and pediatric (1). The item writers were selected based on their clinical expertise and prior/current experiences as educational directors/co-directors. More than 50% of the clinical teachers have teaching experiences of more than 10 years.

The workshop began with an introduction to the concept and purpose of the SJT, followed by a discussion on the steps for developing SJT items and creating a test specification. A test specification, as described by Reed et al. (19), is a document that outlines the specific competency areas/domains intended for assessment using the SJT. This document serves as a reference for item writers throughout the item creation process. Since our goal was to develop SJT items applicable to all residency programs, the first step involved identifying core non-technical/non-cognitive competencies shared across these diverse programs that were suitable for SJT assessment. Following a thorough discussion on the test specification, all item writers—each representing their respective residency programs—agreed on seven key non-cognitive domains to be assessed through SJT. Non-cognitive domains are defined as behavioral attributes such as resilience, empathy, professionalism, that are not related to academic ability but are prerequisites for safe medical practice (20). The agreed non-cognitive domains in this SJT writing workshop include commitment to professionalism, practice-based learning and improvement, systems-based practice, interpersonal skills and communication, teamwork, resilience and adaptability, and patient focus.

Each item writer was tasked with developing five SJT scenarios, each containing six options. The SJTs was a fixed response type SJT where participants would have the rate each response option. Before the writing process, discussion about the non-cognitive domains above were held together with all item writers to achieve similar perceptions.

A template and detailed guidelines (provided as a Supplementary file) were provided to all item writers to guide the SJT writing process including emphasis on how to ensure that SJT items assess non-cognitive attributes, instead of clinical decision making. We recommended item writers to recall a specific past clinical experience and focused on the non-cognitive decision involved in the experiences, not the clinically related. To illustrate this, when item writers recalled a significant event in the operating room, the SJT item should not be about the most appropriate surgical procedure, but on the interprofessional communication between surgeon and the scrub nurses, for example.

Following the initial development phase, the content of the SJT was mapped to specific competency domains to ensure alignment with the intended learning objectives. The number of options and the length of the scenarios were also documented. Furthermore, a qualitative content analysis was conducted using the methods described by Forman and Damschroder (21) to evaluate the items’ appropriateness for measuring non-cognitive aspects during candidate selection. In order to assess the items’ appropriateness, two reviewers independently assessed the content of the scenarios whether or not the scenarios require clinical knowledge to answer. Each reviewer is a medical doctor by training and one of the reviewers has medical education background. Hence all reviewers are familiar with clinical context and able to discern whether or not the items are appropriate to assess non-cognitive attributes since they do not require clinical knowledge and do not involve clinical decision making. The reviewers are also familiar with the item writing guidelines that has been developed for this SJT writing workshop which contains agreed definition of each non-cognitive domain, hence they are able to identify the domains assessed by the scenarios.

This analysis also focused on several important features designed to prevent adverse impacts on candidates, drawing on best practices outlined by Sullivan et al. (22): gender representation, cultural context, language and sensitivity. For instance, the scenarios should not contain slang or jargon that are relevant only to specific groups. The SJT should not portray typical stereotypes of a certain gender, ensuring a balanced reference to both men and women throughout all scenarios. It is also salient to make sure that the SJT scenarios are free of any information that could be considered insensitive or degrading toward certain groups of people or cultural practices.

## 3 Results

A total of 106 SJT scenarios were created. Table 1 maps the distribution of these scenarios across the assessed domains. Most scenarios were designed to evaluate a single domain; however, some overlap occurred: 12 scenarios depicted 2 domains, and 4 scenarios represented 3 domains. The response format was generally consistent, offering 6 options per scenario. The only exceptions were 2 specific scenarios: 1 provided 5 options, and the other offered just a single option. The length of the scenarios ranged from 21 to 145 words.

**TABLE 1:** Distribution of domains of SJT scenarios.

Domains	Number of scenarios
Resilience and adaptability	8
Patient-centered care (focus)	14
Teamwork	5
Interpersonal skills and communication	12
Commitment to professionalism	30
System based practice	12
Practice based learning and improvement	9

Based on the qualitative content analysis, we found no biases or stereotypes related to gender or cultural practices. There was no content or sentences within the SJT scenarios that might be considered degrading or undermining to specific population groups. Overall, in terms of the language, sensitivity and gender, the SJT scenarios developed in this study were considered to be adequate.

Our analysis revealed several findings regarding the appropriateness of using these SJT scenarios to measure the non-cognitive aspects for selecting the most suitable applicants for residency programs. We present some of our findings and sample items below.

First, a few SJT scenarios were inadequate for measuring non-cognitive aspects because they primarily measured clinical decision-making. Consider the following scenario: ‘You request a brain MRI with contrast, as instructed by a senior consultant for your 80-year-old patient with a suspected working diagnosis of intracranial hemorrhage and involuntary movement disorders. Shortly thereafter, you receive word from radiology that the requested test does not require contrast. The reasons given are that there is no indication for contrast, that it would double the examination time, and that the patient’s kidney function is poor. What would you do?’ This scenario requires the test taker to have specific medical knowledge to determine whether to proceed with the contrast MRI or explore an alternative approach. The scenario would be more relevant as an SJT if the focus was on the dispute between the senior consultant in neurology and the one in radiology. Instead, this could be reframed to assess how the resident, as the test taker, navigates between the two opposing recommendations.

Second, some scenarios were too limited in the information provided. Consider the following scenario: ‘During surgery, the nurse gave you the wrong instrument, making it difficult for you to perform the operation and causing a difficult operation. How did you react to the nurse during the operation?’ This scenario needs elaboration with more details that would allow test takers to better evaluate their options and demonstrate their professional judgment. These could be the type of surgery, the type of anesthesia (affecting whether the patient is awake or not), the instrument’s level of importance and the urgency of the surgery, among others. The additional information would strengthen the dilemmas of conflicts depicted in the SJT scenarios, thereby allowing the assessment to better differentiate between candidates’ abilities.

Third, a few SJT scenarios did not portray situations relevant to the clinical setting or residency program. Consider the following scenario: ‘You are involved in an internationally affiliated student organization that will host an annual conference attended by over 500 medical students from around the world. Currently, you are tasked with submitting proposals and conducting presentations to seek funding. One day, you are scheduled to have an audience with the Head of the World Health Organization (WHO) Office in Jakarta regarding your funding request, as well as presentation topics and other support the WHO can provide to your organization. On the same day and at the same time, you are scheduled for coaching to prepare for your summative exam 2, due to your unsatisfactory score on summative exam 1. However, requesting a rescheduling with the WHO is also impossible due to their busy schedules and their position as beneficiaries. What will you do?’ The context depicted in this SJT scenario does not relate to postgraduate residency training. To effectively select candidates based on their non-cognitive attributes, it is recommended to use the clinical workplace setting and the residents’ work and learning processes as the background context for all scenarios.

Fourth, a few scenarios were too heavily contextualized in a particular medical specialty. Consider the following scenario: “You are on frozen section duty, which means performing macroscopic examinations and selecting samples for frozen section. The agreed-upon department policy is that frozen section requests must be scheduled 1 day in advance. Therefore, if the frozen section decision is made intraoperatively, it will only be accepted by 4:00 p.m. That day, there is only one frozen section request scheduled for a 10:00 a.m. surgery, and your consultant has stated that they will not accept frozen section requests after 4:00 p.m. At 3:55 p.m., the frozen section machine is turned off by a technician who then goes to the administration office to handle the day’s examination paperwork. You are about to do some other things and tidy up. At 3:59 p.m., a resident surgeon arrives and brings tissue for frozen section examination at the incision border. You are alone, the machine is off, there are no residents, and you don’t know if your consultant is still there. What will you do?’ Since the SJT was developed for all candidates across all residency programs, scenarios that are too context-specific may advantage certain population groups and be less relatable to others. This lack of general applicability decreases the test’s validity.

## 4 Discussion

This brief research report successfully mapped the content and associated features of SJT items designed for the residency selection process. Overall, the mapping process revealed that most SJT items complied with the specified structure—a vignette, a lead-in statement or question and a set of response options, despite variability in the length of the vignettes and the number of response options. This compliance with the format could be because the item writers first discussed and agreed upon the test specification, based on which the item writing was conducted, before the items were written. This aligns with the first step of SJT development which is a blueprinting process where non-cognitive characteristics that are considered important for workplace performance are discussed and agreed among the subject matter experts (SMEs) (2).

Competent SJT item writers are pivotal to the success of SJT administration. However, many clinician educators are unfamiliar with SJTs. Developing SJT items demands considerable time, effort and a change in mindset because the non-cognitive skills being assessed—such as professionalism—are relatively more difficult to define and operationalize (19). We found several challenges and lessons related to SJT content development process and we discuss these below.

Reed et al. (19) summarized important key points for the SJT item writing process: defining the construct being assessed and recalling real-life critical incidents, specifically those related to affective and behavioral aspects, and using those incidents as a guide in writing the scenarios and deciding on the possible response options. Despite our efforts in providing training and support for item writers, we encountered several persistent problems with the developed SJT items. Precisely, some SJT items were inadequate for measuring non-cognitive aspects. Some scenarios had limited information, hindering candidates from fully considering the breadth and depth of the situation depicted in the scenarios, while some were too heavily contextualized in a particular medical specialty, hence limiting general applicability. There were few scenarios that did not depict any clinical situations at all. Since this SJT was primarily developed for residency program selection to identify candidates with the best non-cognitive attribute potential, it is important to ensure that scenarios accurately and broadly reflect their future workplace.

The current study revealed the challenges of acquiring such specificity and contextualization, despite efforts of guided item writing process, as reflected from the findings where some scenarios are not contextualized enough in the clinical setting or even too contextualized in a certain medical specialty. The findings also show how the amount of information provided needs to be just enough to allow adequate contextualization. According to McDaniel et al. (18), scenarios with specific situational variables may help decrease ambiguity related to the answer. Therefore, for high stakes SJTs it is important to ensure a high degree of contextualization of the scenario (17). Since the SJT developed in this study is meant for selection purposes for residency, scenarios need to be contextualized within healthcare settings to enable assessment on candidates’ behavioral and procedural knowledge on a particular job-related situation.

Regarding content domain distribution, professionalism was the most dominant non-cognitive domain in this study. There were some scenarios containing elements from more than one domain. Soemantri et al. (16) also identified professionalism as one of the most common domains in SJT, alongside effective communication. This dominance may be attributed to the difficulty of differentiating the elements of professionalism (19), potentially leading to some overlaps, and item writers’ preference for the easier ‘route’ of labeling the scenarios as professionalism. Professionalism is also a subject interpreted and practiced quite differently in different settings and contextual backgrounds (23). Ideally, it should be clarified early in the process that each scenario must be mapped to a specific element of non-cognitive attribute, rather than the broad domain (19).

Recommendations for the length of SJT scenarios range from 50 to 100 words to reduce cognitive load (16). However, several scenarios in our study remained beyond this range. While we presented several examples of SJT scenarios and provided guidelines during the workshop, this finding highlighted the importance of continuous support and reminders for item writers during the writing process, especially regarding specific scenario details and item requirements.

Lastly we investigated bias, following Sullivan et al. (22), who emphasized the harmful impacts of bias on content validity. They identified two common biases: language bias and representation bias (which concerns the depiction of members from different subgroups). Language bias occurs when the language or jargon used is more familiar to certain groups than others, favoring the former while unfairly disadvantaging the latter test takers. Representation bias occurs when there is an over- or under-representation of a certain culture or group, including the use of stereotypes, which also disadvantages particular examinees. Our findings revealed that in terms of language and representation, all SJT scenarios and items pose no threats to test validity; they are neutral and show no tendency to degrade certain genders or cultural practices. The language used is common and features regular terminology used in daily conversations, and references to men and women are also balanced across all scenarios.

We realized the limited setting in which our study was conducted, but we argue that our study has shed light on the process for introducing and internalizing the concepts of SJT to subject matter experts who are trained to become SJT item writers. Given the different level of SJT adoption in various countries, we believe the study findings will enable medical educators, who have not applied SJT as a selection tool, to emphasize not only the steps and technicalities of SJT development but also its important nuances, as early as possible in the phase of SJT development. Our study has revealed the complexities of SJT development, noting that many factors can tamper with its validity.

There are several learnings we have gained from this pilot development. For SJT to be a valid and reliable tool for selecting candidates based on non-cognitive attributes, the test items must present dilemmatic situations related to certain non-cognitive attributes to assess candidates’ judgment, not their clinical decision-making abilities and specialized medical knowledge (24). The items should be written in a way that does not favor or disadvantage certain groups of candidates or free from language and representation biases. The recommendation should be very specific on the non-cognitive attribute being assessed, given a wide range and multidimensional nature of non-cognitive attributes. For an SJT to be applicable across an entire range of residency programs, the items should not be too heavily contextualized within a certain medical specialty, as this would unfairly favor candidates applying to that particular specialty.

Our SJT writing workshop yielded promising results, alongside identified challenges and valuable lessons learned. One of our key learnings was that a structured faculty development workshop should start by equipping item writers with adequate understanding of the non-cognitive attributes required by doctors along with relevant cultural nuances, as well as the selection context itself. Not only that, the most prominent challenge we faced in SJT development was establishing its construct validity, ensuring it measures what actually needs to be measured. One way to achieve this is to start with a step by step development of the SJT’s blueprint, followed by specific SJT scenarios and items, along with strengthening the understanding of the construct of non-cognitive attribute, possible biases and specific contextualization of the SJT scenarios. We advocate for early adopters of SJTs as selection methods to pay considerable attention to elements that can threaten the SJT validity during the item writing process such as construct irrelevance, lack of agreement on non-cognitive attributes, and ignorance toward cultural nuances. A faculty development program on SJTs should be conducted continuously to raise further awareness about SJTs and ensure their continued adoption and refinement. Future research could focus on piloting the developed SJT and further establishing its construct validity.

## 5 Conclusion

SJTs are a globally validated tool for selecting candidates based on non-cognitive attributes; however, they are only beginning to gain traction in Indonesia. Our study details the initial phases of SJT development: test specification and an item writing workshop, and has revealed valuable lessons especially for similar SMEs who are inexperienced with SJT development but eager to adopt it. Reflecting on the process and the lessons learned, particularly related to the content and associated features of the SJT, we learned that SJT writing is a complex process, particularly for newly exposed SMEs. Institutions within the early adoption phase of SJTs should start SJT faculty development workshop with at least three principles in mind: first is ensuring mutual agreement of SJTs specification and also the definition and scope of non-cognitive attributes; second, aligning the SJT scenarios and items with the specific construct being measured; and third, incorporating just enough contextual details required for a justifiable non-cognitive judgment. Continuous support and cyclical feedback for item writers are key to the success of SJT writing, as they are essential contributors to the tool’s validity as a selection method.

## Data Availability

The raw data supporting the conclusions of this article will be made available by the author, without undue reservation.
