# Supplementary material for: Incorporating a non-cognitive selection method in the residency program: the complexities of effective situational judgment test item writing
Source: Front Med (Lausanne). 2026 Mar 19;13:1747830. doi: 10.3389/fmed.2026.1747830 (PMC13043418; doi:10.3389/fmed.2026.1747830)
Supplement: Supplementary file 1 [file Table_1.docx]

## **Guidelines for Writing Situational Judgement Tests (SJTs) Items**

### **Characteristics and Format of SJT Questions**

- SJT questions are intended to assess non-cognitive/affective attributes such as professionalism, teamwork, ethics, responsibility, communication, etc.
- Based on the test specification, the current SJTs will assess commitment to professionalism, practice-based learning and improvement, systems-based practice, interpersonal skills and communication, teamwork, resilience and adaptability, and patient focus.
  - Commitment to professionalism include accountability, compassion, integrity, respect and responsiveness
  - Practice-based learning and improvement include self-awareness, properly respond to feedback, continuous reflection on learning and improvement (action plan for learning)
  - System-based practice include working effectively in a healthcare system, cost-benefit analysis, identification and mitigating errors
  - Interpersonal skills and communication include communicate effectively with patients, families, other healthcare professions, and also with the public
  - Teamwork include collaborating effectively within a healthcare team, recognizing other professions’ roles, shared responsibilities
  - Resilience and adaptability include the ability to adapt and persevere in challenging environment or situations
  - Patient focus include acknowledging, recognizing and considering patients’ own views and concerns that should be taken into account when delivering care
- SJT questions will be given to residency candidates applying for entry into a residency program.
- Given that the SJTs will be administered to resident candidates, the questions must not ask anything related to specific specialist knowledge, or require specialist knowledge to be answered correctly.
  - For example, as seen in the sample item below, the scenario does not concern with what is the right medication for the patient hence it does not require specialized medical knowledge, but it assess how the candidates react to the situation where there are two different drugs written in the medical record and prescription list for the same patient. Both drugs were prescribed by the attending specialist and you are the doctor in charge of the night shift. The scenario focuses on how candidates can judge the situation involving the patient and the attending specialist based on the non-cognitive attributes such as communication, patient focus and professionalism.
- The SJTs should use clinical contexts relevant to medical graduates (General Practitioners) or other situations/conditions relevant to a doctor. This can include learning activities in medical or specialist programs, provided they do not require specialist medical knowledge to answer.
- The SJT format is RATING, where there is one scenario and each scenario consists of 4–6 answer choices or items. Participants must provide a value/rating between 1–4 for each item/choice:
  - 1 = Very appropriate
  - 2 = Appropriate but not ideal
  - 3 = Inappropriate but not bad
  - 4 = Very inappropriate

**Sample item**

| Instructions for test participants | After reading the scenario below, the test participant must provide a rating/score regarding the appropriateness level of each answer choice for the given scenario. |
| --- | --- |
| Rating | **1** = very appropriate, **2** = appropriate but not ideal, **3** = inappropriate but not bad, **4** = very inappropriate |
| Scenario | While you are on night duty in the ICU, you are studying the medical records of a patient with a severe heart condition being treated in the ICU. You notice in the patient's follow-up notes in the medical record that the attending specialist wrote down the administration of **dopamine** for the patient. However, when looking at the prescription slip written by the specialist, it turns out the drug prescribed was **dobutamine** at the same dose as the dopamine dose. This dobutamine has actually been administered to the patient since 2 hours ago. Given the patient’s condition, you know that the patient should receive dopamine instead of dobutamine. Currently, the patient is in relatively stable condition.  What would you do in the situation above? |
| Response item | |
| A | Ask the nurse which medication should actually be given to the patient. |
| B | Change the patient's medication to a dopamine infusion |
| C | Leave the medication administration as it is until the morning rounds with the specialist the next day |
| D | Immdiatenly contact the specialist via telephone to ask about the medication that should be given |
| E | Inform the patient about the medication error |

**Item template**

| Non-cognitive domain |  |
| --- | --- |
| Instructions for test participants | After reading the scenario below, the test participant must provide a rating/score regarding the appropriateness level of each answer choice for the given scenario. |
| Rating | **1** = very appropriate, **2** = appropriate but not ideal, **3** = inappropriate but not bad, **4** = very inappropriate |
| Skenario | ……. |
| Response item | |
| A | …. |
| B | …. |
| C | …. |
| D | …. |
| E | …. |
| F | …. |
